# Primary central nervous system vasculitis: an update

**DOI:** 10.1007/s00415-026-13727-y

**Published:** 2026-03-03

**Authors:** Claire Rice, Neil Scolding

**Affiliations:** Institute of Clinical Neurosciences, University of Bristol, Bristol, England

**Keywords:** CNS vasculitis, CNS angiitis, Cerebral vasculitis

## Abstract

Primary CNS vasculitis (PCNSV) is uncommon, difficult to diagnose, and potentially fatal or at least likely to cause major and persistent neurological deficits, and yet appears highly treatable (although controlled clinical trials remain to be done). Here, we summarize recent advances in the recognition and diagnosis of PCNSV, the crucial role of biopsy and histopathological confirmation of the diagnosis, and currently recommended approaches to treatment. We also sign post areas of potentially fruitful future collaborative study that might advance this difficult disease.

## Introduction

Primary CNS vasculitis (PCNSV) presents the neurologist with a rare level of clinical jeopardy, a consequence of the unusual and unsettling combination of being uncommon, potentially life-threatening, and yet highly treatable (though a further irony is that there still remains a complete absence of controlled therapeutic trials in PCNSV). Add to this the absence of any reliable diagnostic clinical features, the presence of almost innumerable neurological mimics, and the continuing lack of any diagnostic investigation short of brain biopsy, and the unease felt by most clinicians when confronted with a potential case is readily understood.

Cravioto and Feigin first described vasculitis confined to the CNS over 65 years ago, delineating the classical histopathological features of the disorder, definitively describing it as a “diffuse disorder of the central nervous system with some focal accentuation” [1]. Calabrese and Mallek’s landmark study three decades later provided a lasting account of the clinical features, summarized the angiographic changes, and emphasised the recommendation for high dose corticosteroids and cytotoxic drugs, specifically cyclophosphamide, in therapy [2].

CNS vasculitis is characterized by inflammation within the wall of CNS blood vessels associated with destructive changes, occlusion and infarction. It is suggested that three histopathologic patterns of vasculitis may be distinguished, granulomatous, lymphocytic, and necrotizing, though the variability associated with ‘skip’ lesions makes it difficult to know whether multiple types might occur in a single patient [3]. In ‘secondary’ CNS vasculitis, the CNS becomes involved in a systemic vasculitic illness, such as microscopic polyarteritis, or systemic rheumatoid; these disorders will not be considered here. In primary (or ‘isolated’) CNS vasculitis, there is little or no overt systemic inflammation.

## Clinical features

Many neurological features can occur, including headache, encephalopathy, cognitive change, generalized seizures, and focal neurological abnormalities, and the course may be acute or subacute, chronic, or relapsing and remitting [2, 4–9]. Curiously and thus far inexplicably, unilateral disease is well-described although unusual (in which, interestingly, even biopsy-proven disease seems not uncommonly to have unremarkable angiography, [10] likely related to the size of vessel affected and the resolution limits of angiography). Systemic features of inflammatory disease – fever, night sweats, oligoarthropathy, etc., may be present.

Notwithstanding the absence of any pathognomic clinical picture, three clinical patterns of presentation have been identified [11, 12] :-

- an acute or subacute encephalopathy, sometimes progressing to coma;
- a picture *superficially* resembling multiple sclerosis – a relapsing-remitting course, perhaps with an optic neuropathy and brain stem episodes, *but* also with other features less common in multiple sclerosis – seizures, severe headaches, encephalopathic episodes, or stroke-like episodes;
- intracranial mass lesion[s] [13] with headache, drowsiness, focal signs and raised intracranial pressure.

These are far from specific to PCNSV but might usefully raise suspicion when an alternative explanation is not obvious.

## Investigation

No blood, cerebrospinal fluid (CSF) or imaging investigations are specifically diagnostic of PCNSV, though many are abnormal. Anaemia (normochromic), neutrophilia, and raised plasma viscosity are common. The CSF is abnormal in approximately 75% of cases, and brain MRI in over 90% (not, notably, 100%), but neither shows changes that are unique to CNSV [14]. Efforts to develop specific MRI-based approaches for diagnosing cerebral vasculitis continue, including vessel wall imaging [15, 16] but none has yet withstood rigorous MR-neuropathological correlation [17, 18]. Still, biopsy-based correlative studies are beginning to advance the MRI recognition and diagnosis of PCNSV; abnormalities of vessel calibre, the presence of ischaemic insults of varying ages, a lack of respect for vascular territories, and the presence of some enhancement and inflammatory changes within the vessel wall can all inform MR scanning approaches [19].

Therefore, the principal role of blood tests, CT of chest, abdomen and pelvis, CSF examination and MRI scanning (all more or less mandatory whenever PCNSV is suspected) is to exclude alternative disorders, often inflammatory or malignant, sometimes disclosing clinically covert and accessible biopsy targets. Slit-lamp ophthalmoscopy [20] and fundus fluorescein angiography (to help exclude, for example, Susac’s disease), and also whole-body CT-PET scanning are likewise important.

*Cerebral angiography*, either ‘formal’ (i.e., catheter contrast/digital subtraction), CT-based and/or MR-based, may be abnormal. Typical vasculitis changes include multifocal segmental narrowing combined with localized dilatation or beading. Technical improvements in MR- and CT- notwithstanding, contrast/digital subtraction angiography remains more specific and sensitive [21–24] (but does carry a very small risk of stroke). However, neither the sensitivity nor the specificity of catheter angiography in PCNSV are above 25–35% [2, 6, 14, 25–28] (despite which, many published ‘PCNSV’ studies continue to depend on catheter angiography in their diagnostic confirmation). Alternative causes of comparable angiographic changes include atherosclerosis, subarachnoid haemorrhage, migraine, trauma, hypertension, infections, radiation vasculopathy, reversible cerebral vasoconstriction syndrome (RCVS) and recreational drug use. [29, 30] It should, of course, also be noted from these figures that a normal angiogram does not exclude PCNSV.

*Cerebral biopsy* currently remains, in our view, the gold standard for the diagnosis of PCNSV, despite both the potential dangers of such an invasive procedure, and the less than perfect sensitivity – the frustrating neuropathological finding of only ‘non-specific change’, ‘end-stage tissue damage’ or ‘gliosis’. But the risks, particularly of death or serious or permanent morbidity have been repeatedly and objectively shown to be very low, many studies suggesting a procedure-related mortality of zero [25, 31–33]. Even brain stem and spinal cord biopsies may be less hazardous than previously assumed [34]. And these risks must be seen in the context of the life-threatening nature of PCNSV and many of its clinical mimics, and of the potentially hazardous therapies that may subsequently be employed [35, 36]. Current evidence suggests a sensitivity of between 50 and 70% [2, 6, 25, 31] – although from a practical clinical perspective, around 75% of patients receive a clear diagnosis (vasculitis *or* another specific disorder) that directly informs management [25, 31, 35]. That the alternative diagnosis emerging is often infective emphasizes in particular the danger of opting for empirical treatment with immunosuppressants; while failing (again through omitting biopsy) to disclose another common mimic, cerebral lymphoma, could also deny the patient of a potentially treatable diagnosis. A large meta-analysis suggests that frame-based and frameless biopsy do not differ in diagnostic yield, morbidity, and mortality [37].

Finally (though this in principle should play no role in any individual patient decision), advancing our clinical understanding of cerebral vasculitis depends in various ways on histopathological analysis. Firstly, clinical ‘PCNSV’ studies can only be reliable and accurately informative if restricted to cases of definite disease. Given the very poor sensitivity and specificity of angiography, this means exclusively biopsy-proven PCNSV (though this, not uncontroversially, would exclude probably the majority of published studies, some 75% of which lack histopathological proof, [38] since such studies must include significant numbers of non-vasculitic cases as ‘PCNSV’). Less provocatively, we now know, through biopsy studies, that PCNSV comprises a spectrum of specific disorders, including *Aβ-related Angiitis (ABRA)*, *Small Vessel Inclusion Body CNS Vasculitis* (usually affecting only children) and *Hypereosinophilic CNS Vasculitis* [39–41]. Other specific entities must surely emerge in the future; and careful, biopsy-based studies will also allow greater insights into PCNSV, including diagnosis, prognostication and potential treatment stratification [19, 42–44] (helping to confirm, for example, early clinical impressions that ABRA-related CNS vasculitis may have a better prognosis than PCNSV [44]) – though only if biopsies continue to be performed, with expert analytical subsequent tissue examination.

## Diagnostic criteria for PCNSV

The extremely limited sensitivity and specificity of an angiogram-based ‘diagnosis’ of vasculitis is well-illustrated first by one particular study at a (major) US academic hospital, in which brain biopsies were performed in 14 patients with clinical and angiographic features considered diagnostic for PACNS, none being found to have vasculitis on histopathological examination [45]; and second, by considering that, since the positive predictive value of cerebral angiography in this context is less than 30% [28], patients with ‘typical’ vasculitic changes could arguably be said to be statistically more likely to have an alternative disorder than PCNSV [14, 35, 45, 46]. Recent work has confirmed that there are no reliable ways of distinguishing amongst patients with suspected PCNSV, those who turn out to have biopsy-proven disease [47].

While from a practical clinical perspective it is occasionally necessary to ‘label’ a patient as having PCNSV without histological proof, and to treat accordingly, we have argued that it is much harder to defend the still common rule that cases lacking biopsy proof can still be definitively termed PCNSV in the published, peer-reviewed literature [48]. Others have also asserted that published case reports, case series, or reviews should only include patients with histological confirmation, [14, 46] though major studies continue to appear that describe cohorts where fewer than 65% patients have biopsy-proven disease [49, 50] (or even under a third [43]).

In consequence, we proposed new binary diagnostic criteria (Table 1) – 'possible’ or ‘definite’ PCNSV, depending on the presence or otherwise of biopsy proof [48]. There is no ‘probable’ category; cases without biopsy confirmation are no more than ‘possible’

**Table 1:** Proposed criteria for the diagnosis of CNS vasculitis (CNSV) [48]

Definite	
*Clinical presentation suggestive of CNSV with exclusion of alternative possible diagnoses and of primary systemic vasculitic syndrome*	
PLUS the presence of positive CNS histology, i.e., biopsy or autopsy showing CNS angiitis (granulomatous, lymphocytic, or necrotizing), including evidence of vessel wall damage.	

Possible	
*Clinical presentation compatible CNSV with exclusion of alternative possible diagnoses and of primary systemic vasculitic syndrome*	
PLUS laboratory and imaging support for CNS inflammation (elevated levels of CSF - protein and/or cells, and/or the presence of oligoclonal bands and/or MRI evidence compatible with CNSV), with angiographic* exclusion of other specific entities	
BUT without histological proof of vasculitis.	

## Treatment

As mentioned above, PCNSV treatment has no evidence base in clinical trials, and recommendations are based solely on evidence from therapeutic trials in renal and/or rheumatological vasculitis (where the diagnosis can be robustly determined) combined with expert experience. High dose corticosteroids and cyclophosphamide are the mainstay of induction therapy [11, 29, 51–55], followed by maintenance with less toxic immunosuppressants such as azathioprine, methotrexate or mycophenolate. In systemic vasculitis, azathioprine or methotrexate appear superior to mycophenolate in maintaining remission [56]. There are reports of the potential efficacy of rituximab, [57] but again these are often based on cases lacking histopathological verification. There is evidence from systemic vasculitis trials that combining rituximab with cyclophosphamide may safely reduce patient exposure to corticosteroids. [58, 59]

## Summary

Advances in neuroimaging and biomarker identification are likely in the future to improve time to diagnosis and monitoring of PACNS with consequent benefit in terms of clinical outcomes, but, in our view, histological confirmation of the diagnosis remains key to their validation. We acknowledge that, in clinical practice, empirical treatment may be justified/necessary in some circumstances, but we recommend greater emphasis on studies reporting cases of biopsy-proven PACNS and that future treatment trials in PACNS should be restricted to those with a histologically-confirmed diagnosis. Given the rarity of the condition this will, necessarily, require co-ordination of clinical networks both nationally and internationally to develop a robust clinical research platform including deep clinical phenotyping, biomarker discovery and validation, as well as clinical trials with the ultimate aim of developing personalized approaches to PACNS diagnosis and treatment with improved clinical outcomes.
